# Adjunctive use of modified *Yunu*-*Jian* in the non-surgical treatment of male smokers with chronic periodontitis: a randomized double-blind, placebo-controlled clinical trial

**DOI:** 10.1186/s13020-016-0111-z

**Published:** 2016-09-20

**Authors:** Kwan-Yat Zee, Pui Sze Chan, Johnson Chun Sing Ho, Stanley Man Lung Lai, Esmonde Francis Corbet, Wai Keung Leung

**Affiliations:** Faculty of Dentistry, The University of Hong Kong, Hong Kong, SAR China

## Abstract

**Background:**

*Yunu*-*Jian* (YJ) is a Chinese medicine (CM) *heat* purging formula, which is used to reduce *wei huo* (*stomach*-*heat*, SH) and enrich *shen yin* (*kidney*-*yin*, KY). This formula is also commonly used to manage diabetes mellitus and gum/oral inflammation. The activity of YJ can be modified or refined by the addition of other CM herbs and/or minor changes to one of its five key ingredients. The aim of this study was to evaluate the adjunctive use of modified YJ (mYJ) or YJ containing additional osteoblast-stimulating and inflammation-modulating CM herbs in the non-surgical periodontal treatment of smokers with chronic periodontitis in a randomized, double-blind, prospective, placebo-controlled study.

**Methods:**

Healthy adult male smokers with untreated chronic periodontitis who showed CM syndrome of SH and KY deficiency (KYD) whilst attending a dental teaching hospital from October to December, 2005, were invited to participate in a randomized double-blind, placebo-controlled clinical trial. The trial itself involved the once-daily oral administration of a placebo or mYJ for 3 months as an adjunct to non-surgical periodontal therapy. Several periodontal parameters, including radiographic alveolar bone density, were measured by computer-assisted densitometric image analysis (CADIA) on selected sites, and CM signs of SH and KYD were followed from their baseline values to various time points up to 12 months or the end of study.

**Results:**

Twenty-five smokers (consumed 25.0 ± 15.3 smoking-pack years, ranged 7.5–80; aged 46.3 ± 6.8 years) with periodontitis and SH and KYD were recruited (Placebo, n = 14; mYJ, n = 11). All of the participants showed good tolerance towards the CM recipe. All of the periodontal parameters had improved after 12-month follow-up, and no statistically significant differences were detected between the control group and test group, except for the higher CADIA values observed compared with the baseline at 12 months for test sites (*P* = 0.025). 4/3/3 test vs 14/13/13 control participants had persisting SH and KYD at 6, 9 and 12 months (*P* < 0.001), respectively.

**Conclusions:**

The adjunctive use of mYJ preserved the post-treatment increases in the radiographic alveolar bone density at the study sites and led to an overall improvement in SH and KYD compared with the controls.

*Trial registration* HKU Clinical Trial Register, HKCTR-1848 (www.hkuctr.com/Study/Show/3acbf983831244d29d50b543540bf6e9)

**Electronic supplementary material:**

The online version of this article (doi:10.1186/s13020-016-0111-z) contains supplementary material, which is available to authorized users.

## Background

Periodontal disease is an infectious disease that affects the periodontium of susceptible hosts, and is characterized by the inflammation and destruction of the surrounding tooth-supporting tissues [1]. Approximately 20 % of the adult population of Hong Kong has a probing pocket depth (PPD) ≥6 mm [2]. Although bacteria play a critical role in the life cycle of this disease, they are not solely responsible for the onset of this disease, with host factors playing an important role in the outcome of this disease [3]. Smoking is one of the greatest risk factors for periodontitis [4, 5], and is associated with an increased risk of periodontal attachment loss [2, 6]. Although routine non-surgical periodontal therapy has been shown to be effective for the treatment of chronic periodontitis in smokers, smokers tend to exhibit more residual periodontal pockets [7–9] than non-smokers. Smokers also show a greater tendency towards progressive radiographic alveolar bone loss despite post-treatment periodontal maintenance care [10].

In the conceptual context of Chinese medicine (CM), periodontal disease is mainly categorized as *ya xuan* (gaping gums), *ya lou* (leaking gums) and *ya nu* (bleeding gums) [11, 12]. The recommended treatment for this disease involves the mechanical removal of deposits from the affected teeth [13, 14]. The use of herbal medicine recipes has also been suggested to rectify the disease-associated imbalance in the human body along the four major philosophical frameworks, including attention to poor oral hygiene, *wei huo* (*stomach*-*heat*, SH), *shen yin xu* (*kidney*-*yin deficiency*, KYD) and weakness in *qi* and *xue* (*blood*) [15]. The concept of CM dictates that the *kidney* dominates the *gu* (*bone*) and *sheng sui* (*create marrow*) and that the *kidney* therefore also dominates the teeth, whereas the *pi* (*spleen*) and *stomach meridians* pass through the teeth and gum. Accordingly, periodontal disease could therefore be a problem associated with unwanted changes to the homeostasis in the *spleen* and *stomach* or *kidney* [14]. From the perspective of CM, smoking would not only affect the lungs and airways, but would also fire up the *qi*, destroy blood and consume *yin* fluids [16].

Several CM formula have been used to treat *ya xuan, ya lou* and *ya nu*, including *Radix Achyranthis bidentatae* (*Huai Ni Xi*), *Rhizoma Drynariae* (*Gu Su Bu*), *Radix Ophiopogonis* (*Mai Men Dong*), *Cortex Phellodendri* (*Huang Bai*) and *Radix et Rhizoma Rehmanniae* (*Sheng Di Huang*). All of these recipes have been reported to attenuate in vivo inflammation, enhance immune function and/or modulate bone homeostasis [17, 18]. *Rhizoma Drynariae* and *Rehmannia glutinosa* (*Shu Di Huang*) has also been reported to exhibit therapeutic effects towards bone fracture healing, including the induction of proliferation and osteogenic differentiation of human bone mesenchymal stem cells [19, 20]. *Rhizoma Drynariae*, which contains naringin as its main effective component, is one of the most widely investigated of all of these herbal preparations [20].

*Yunu*-*Jian* (YJ), which is also known as Jade maiden/women decoction, Fair maiden decoction or *Rehmannia* and *gypsum* combination, is a CM *heat* purging formula used to reduce *stomach*-*heat* (SH) and enrich *kidney*-*yin* (KY) [13]. This formula is also commonly used to manage diabetes mellitus and gum/oral inflammation. In terms of the different concepts of CM, the regular smoking of cigarettes leads to the *burning* of the *stomach* and *fei* (*lung*) [21], placing the affected individual at a higher risk of experiencing *ya xuan* due to SH. The activity of YJ can be modified or refined by the addition of CM herbs and/or minor changes to one of its five key ingredients. The aim of this study was to explore the adjunctive effect of using a specifically modified YJ (mYJ) formulation (Table 1) to target SH and KYD in male smokers with chronic periodontitis. In terms of its modification, *Rhizoma Drynariae, Cortex Phellodendri* and *Radix Puerariae* (*Ge Gen*; to promote circulation and increase blood flow) were added to YJ, whereas *Rehmannia glutinosa* was replaced with *Radix et Rhizoma Rehmanniae*. Overall, this study applied the CM *ya xuan* treatment philosophy to achieve host modulation in contemporary periodontal therapy [18].

**Table 1:** Composition of modified *Yunu*-*Jian* (mYJ) and placebo

Chinese name	Pharmaceutical name	g/day^a^
*Yunu*-*Jian*	*Rehmannia* and *gypsum* combination	
*Shi Gao* ^b^	*Gypsum Fibrosum*	25
*Sheng Di Huang*	*Radix et Rhizoma Rehmanniae*	20
*Mai Men Dong* ^b^	*Radix Ophiopogonis*	18
*Zhi Mu* ^b^	*Rhizoma Anemarrhenae*	15
*Huai Niu Xi* ^b^	*Radix Achyranthis Bidentatae*	15
*Gu Sui Bu*	*Rhizoma Drynariae*	15
*Huang Bo*	*Cortex Phellodendri*	10
*Ge Gen*	*Radix Puerariae*	15
Placebo		
*Ku Gu*a	*Momordica Charantia*	20

^a^Weight equivalent of the raw ingredient per preparation

^b^Together with *Rehmannia glutinosa (Gaetn.) Libosch. Ex Fisch. Et Mey.* (20 g raw ingredient per preparation) constituted the original *Yunu*-*Jian* recipe

In recent years, over-the-counter CM preparations such as *Guchi*-*Jianzhou* granules, *Guchi* pills (or *Guchi Wan*), *Guchi* extract and *Yazhou Baidu* powders have been developed to aid in the management of chronic periodontitis [18]. Despite the increasing availability of potential adjunctive agents, there have been very few properly designed CM-periodontal clinical trials or reports pertaining to the correct clinical usage of these preparations or formulae.

Non-surgical mechanical therapy can be effective for the treatment of periodontitis [22]. Smokers, however, have been repeatedly shown to experience inferior healing responses following non-surgical periodontal therapy [7, 9]. Clinicians have attempted a variety of approaches, including adjunctive antibiotics [23], toothpastes containing triclosan/copolymers in an un-blinded fashion [24], and Er:YAG lasers, in place of regular mechanical therapy [25] to enhance or augment non-surgical periodontal healing in smokers. Unfortunately, however, none of these efforts have produced satisfactory results.

This study aimed to evaluate the adjunctive use of mYJ (Table 1) for the non-surgical periodontal treatment of male smokers with chronic periodontitis in a randomized, double blind, prospective, placebo-controlled study. Middle-aged male smokers who had been diagnosed with CM syndrome of SH and/or KYD, but were otherwise healthy, were recruited into this trial. Full-mouth clinical treatment responses were measured, together with radiographic treatment outcomes at selected sites, which were measured by computer-assisted densitometric image analysis (CADIA) and gingival crevicular fluid (GCF) volume. Digital subtraction radiography (DSR) was used to supplement the periodontal healing response assessment as described before [26], which is considered as a sensitive follow-up protocol for alveolar bone healing after periodontal treatment [27, 28]. General CM signs of SH and KYD were recorded over a 12-month study period. Standard clinical and radiographical periodontal parameters were followed throughout the clinical trial to provide an indication of the reduction in the periodontal inflammation (reduction of bleeding on probing or BOP), resolution/remodeling of the diseased periodontium (reduction of PPD) and bony repair (increase in radiographic periodontal bone density) [29]. As the monitoring of changes in the key CM syndrome of participants during a CM clinical trial was deemed necessary [30], and the current study was conducted accordingly.

## Methods

### Ethics

The research protocol was approved by the Ethics Committee, Faculty of Dentistry, The University of Hong Kong (HKU) (1/8/12d) (Additional file 1). Interested individuals were verbally briefed about the nature and details of the study with the aid of written explanatory notes covering for any potential risks or benefits from participating. They were given 1 week to think about the project and were advised to discuss participation with family members, referring doctors or dentists before returning to the Periodontology Clinic, HKU and expressing whether to part-take the study or not. On both occasions they were invited to ask as many questions as they liked and the person responsible (PSC) took as long as needed to answer all issues raised. Written informed consent (Additional file 1) was obtained from all participants.

### Study design

**Fig. 1:**
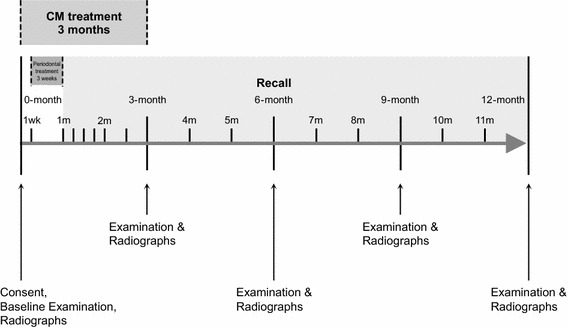
Study design

This study was conducted as a prospective, randomized, double-blinded, placebo-controlled clinical trial. Participants were randomized into control and test groups as follows. All enrolled individuals diagnosed with SH and/or KYD received non-surgical periodontal therapy over four appointments (i.e., four times, each time a quadrant or upper/lower, left/right quarter of mouth treated) completed in a 3-week period. All of the participants received an oral once-daily dose of mYJ or placebo for 3 months, followed by regular post-treatment recalls for up to 12 months (i.e., the end of study or 11 months post-periodontal treatment completion) (Fig. 1). A placebo powder (total 25 g) with a similar physical appearance and taste to mYJ was prepared using several inactive components, including starch, glucose and *Momordica charantia* or *Ku Gua* (2 g), which was used as a naturally bitter component.

### CM formula preparation

A mYJ, which contained eight Chinese medicinal ingredients, was used (Table 1). The mYJ or placebo were produced in dissolvable powder form by a registered CM manufacturer in Hong Kong (Eu Yan Sang [Hong Kong] Limited). In brief, correct amounts of all raw materials were boiled together in water to extract their ingredients. The aqueous extract was then concentrated, dried, and crushed into fine powder. The correct amount of powder was then packed into an individually sealed plain aluminum sachet (25 g). A placebo powder (total 25 g) was packed in the similar aluminum sachets as the active recipe. The placebo or active sachet was coded as PH001 or PH002, respectively by the manufacturer. The identity of the sachets was kept by one of the authors (KYZ) who did not participate in the collection and analysis of the data. Contamination screening against heavy metals, pesticides, and aflatoxins in both the active and placebo sachets was performed by an independent laboratory to ensure safety before human consumption. All CM agent preparation and toxicity testing were completed by September 2004.

### Sample size determination

This clinical study targeted males with chronic periodontitis, who were otherwise healthy (self-assessment), except for the fact that they had been diagnosed with SH and/or KYD syndrome. Changes in the CADIA value were considered as the primary dependent variable. The sample size for this study was computed according to the following formula [31]:

$${\text{n}} = \,\left[ { 2\sigma^{ 2} \left( {{\text{Z}}_{\alpha / 2} + {\text{ Z}}_{\beta } } \right)^{ 2} } \right]/\delta^{ 2}$$

where n = sample size, σ^2^ = variance, δ = expected difference, Z_α⁄2_ = 1.96 if α = 0.05, Z_β_ = 0.842 if power = 80 %.

In a study involving non-smokers from the same local population, participants with chronic periodontitis showed changes of 60 ± 25 in their CADIA values at 12 months after non-surgical therapy [26]. We assumed that the SD would be the same for smokers as it was for the non-smokers in this previous study. We also assumed that there would be an increase of 30 units (expected difference or δ) in the CADIA value at the diseased sites between smokers taking the placebo compared with those taking mYJ. Based on these assumptions, the above formula indicated that 10.9 or 11 participants would be required in each group to enable a difference of 30 units in the CADIA values to be detected.

### Participant selection and screening

New male participants attending the Reception Clinic, Prince Philip Dental Hospital (PPDH), Faculty of Dentistry, The University of Hong Kong and satisfying the inclusion criteria were recruited to participate in the study. The target sample size was at least 14 participants each for control and treatment group, to allow for retention of 11 individuals in each group at 12 months. For inclusion, participants had to be reportedly free of systemic disease, and displaying the following features: (1) 36- to 64-years-old ethnic Chinese with untreated chronic periodontitis; (2) smokers with a current consumption of ≥10 cigarettes per day for at least 10 years and expressing no interest in quitting smoking in the coming 12 months; (3) at least 16 standing teeth, with at least 1 tooth having PPD ≥5 mm in each quadrant, excluding the third molars; (4) radiographic evidence of alveolar bone loss at a proximal site up to one-third of root length in at least two teeth per quadrant; and (5) diagnosed as showing signs of SH and/or KYD CM syndrome (Additional file 2: Table S1). Participants were excluded if the initial interview revealed: (1) systemic disease history; (2) history of taking systemic antibiotics in the preceding 30 days; (3) history of any dental treatment, other than oral hygiene instructions, in the preceding 30 days; (4) known hypersensitivity to any Chinese medicine in particular to any ingredients of mYJ or control recipe used in this study; (5) history of rheumatic fever or other conditions for which antibiotic prophylaxis prior to periodontal examination or invasive dental treatment might be advisable; (6) undergoing concurrent dental therapy such as orthodontic treatment.

Thirty two individuals satisfying the inclusion criteria were recruited by the end of a 3-month period (October to December 2005) while the whole study was finished approximately 12 months later.

### Participant management and non-surgical mechanical periodontal treatment

**Fig. 2:**
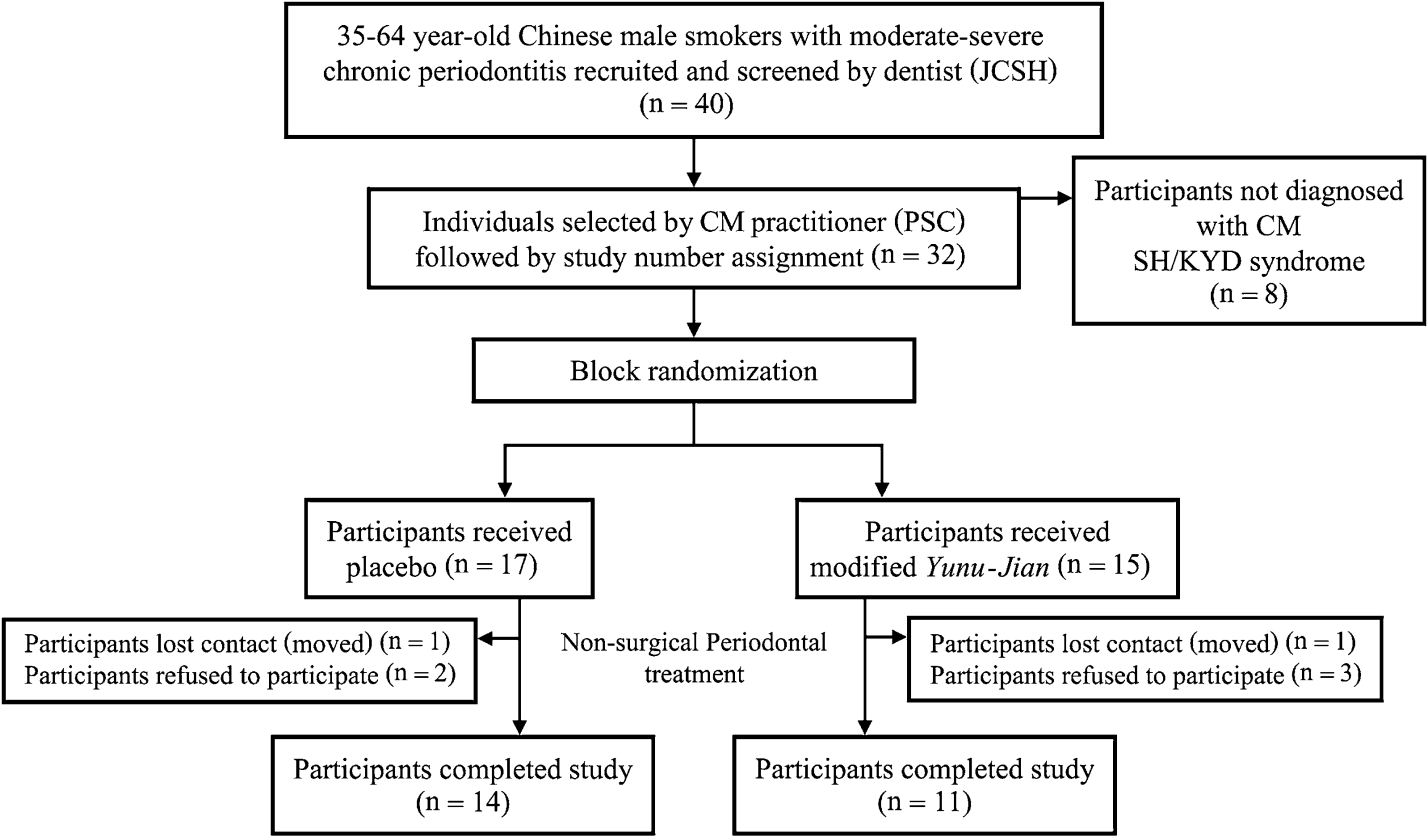
Flow of participants through each stage of the randomized trial

This clinical study was carried out at the Periodontology Clinic, PPDH, Faculty of Dentistry, HKU, China. A member of the research team (JCSH) evaluated the dental eligibility of male smokers presenting at the clinic with untreated chronic periodontitis. Male smokers satisfying the eligibility requirements were asked whether they would like to participate in this study, and the CM eligibility of those who accepted the invitation was confirmed by PSC. Eligible participants were then assigned a numerical personal identification code (study number) as a personal identifier to be used throughout the study (Fig. 2). JCSH then followed up with all of the eligible participants to ensure that all of the necessary pre-treatment processes had been conducted. Emergency treatments such as extractions, caries stabilization and initial endodontic therapy were completed prior to the commencement of the non-surgical periodontal treatment trial.

After the required number of participants had been secured, receptionists at the Periodontology Clinic, PPDH, were instructed by KYZ via a concealed envelope, which was prepared prior to the recruitment process, to arrange appointments with all of the participants. Each envelope also included a predetermined table with a study number against randomized blocks indicating sachet allocation. Critically, the attending receptionists were completely unaware of the contents of the different sachets. All of the participants signed an informed consent form at their first appointment and were subsequently presented with seven designated sachets with specific instruction to take one sachet per day for the week prior to the commencement of the non-surgical periodontal therapy. On the first day of the non-surgical periodontal therapy, each participant was presented with 23 identical sachets to those that they had already received. The participants were subsequently presented with 30 more of the same sachets at onw and 2 months after the commencement of the non-surgical periodontal therapy. KYZ was responsible for concealing the details of each participant’s allocation, whilst simultaneously refrained from contact with any of the participants, receptionists or clinicians involved in the study until it reached completion.

Each participant was instructed to start consuming the content of the allocated sachets 1 week before starting the non-surgical periodontal therapy process. The participants were instructed to dissolve the powder in each sachet in 200 mL of hot water and drink the resulting solution while it was still warm at the beginning of each day. Each participant was issued with a total of 90 sachets. Self-completion compliance sheets were given to each participant, when CM sachets were issued, to be completed at predefined intervals, including (1) within the 1 week pre-periodontal treatment period; (2) from day 8–day 30 after commencement of the periodontal treatment; and (3) over the second and third month study period. These forms were provided to encourage proper CM medication consumption and to monitor self-reported compliance. Participants had to sign the compliance sheets to confirm their medication consumption and return the sheet, together with any untaken CM medication, according to the schedule set. On each compliance sheet, there was a section for participants to fill in the date, time and type of any suspected adverse effect experienced. Two phone numbers (accessible to PSC or JCSH anytime during the study period concerned) were written on the compliance sheet in case any participant wanted to contact the investigation team.

The receptionists at the Periodontology Clinic were instructed to arrange the non-surgical periodontal treatment appointments (4 visits) within a 3-week period for all of the participants. The appointments were subsequently delivered by JCSH, who was not involved in the clinical data collection. All of the participants received the same non-surgical periodontal treatment, together with the same oral hygiene instructions regarding brushing and interdental cleaning, followed by quadrant-wise scaling and root surface debridement under local anesthesia.

Recall appointments were scheduled once every week for the first month after the periodontal treatment, with reduced frequencies thereafter (i.e., once a week at month 2; once every 2 weeks for month 3, and once every month until the end of study). Oral hygiene reinforcement and motivation were provided as necessary. Supra- and sub-gingival debridement was performed if needed during the recall appointments. Clinical and radiographic examinations were carried out at the 3-, 6-, 9- and 12-month recall visits.

Any residual periodontal problems detected at the conclusion of the study at the 12-month recall, namely any sites with residual PPD >5 mm, were followed-up with an appropriate periodontal treatment process (e.g., re-root planing or access surgery), which was arranged and delivered without delay. All of the participants were again reminded of the deleterious effects resulting from their continued smoking and were advised to quit smoking at all of the recall appointments and at the end of the clinical trial.

### Clinical examination

Several clinical parameters were recorded at the baseline, as well as being recorded at 3, 6, 9 and 12 months after enrollment. All of the clinical examinations were performed by one calibrated examiner (SMLL).

The PPD and probing attachment level (PAL) measurements were recorded at six different sites for each tooth, excluding the third molars (i.e., the mesio-buccal, mid-buccal, disto-buccal, mesio-lingual, mid-lingual and disto-lingual sites). Custom-made poly-ethylene occlusal stents were fabricated for each participant as reference guides to ensure the reproducibility of the probing sites and for measuring the probing attachment level throughout the study using a manual periodontal probe (PCP-UNC 15, Hu-Friedy probe^®^, Chicago, IL, USA). The presence of any plaque was recorded dichotomously as the presence or absence of plaque according to the detection of plaque deposits determined by running the tip of the periodontal probe along the tooth surface at the gingival margin of each site. BOP was designated as positive if bleeding occurred within 10 s of periodontal probing.

Radiographic examinations were carried out at the two deepest proximal single-root tooth-sites in each participant. The two deepest non-neighboring sites (PPD ≥ 5 mm) of the mouth on two individual teeth with well-defined angular defects were selected as described previously [26]. The sites were selected initially from the panoramic oral radiograph taken as part of the PPDH admission screening procedure and subsequently confirmed by paralleling periapical radiographs. The gingival crevicular fluid volumes of these sites were also recorded (last paragraph of this section).

Radiographic examinations for following up any changes in alveolar bone by standardized DSR were carried out as described by Woo et al. [29] and Lai et al. [26]. Briefly, after selecting suitable sites for follow up, a custom-made acrylic bite block attached to a Rinn XCP film holder (Rinn Corporation, Elgin, IL, USA) was constructed for each of the selected sites to produce standardized reproducible periapical radiographs for digital subtraction [32]. Rectangular and long cone techniques were used with a radiographic film requiring a short exposure time (Kodak Insight, Eastman Kodak Co., Rochester, NY, USA) to minimize the level of radiographic exposure to the participant. The X-ray cone was also modified to allow for the rigid attachment of the bite block to the cone. As a consequence, the angulations between the X-ray source, the object and the film were reproducible. All periapical radiographs were taken using the paralleling technique with the same X-ray machine (GE 700, General Electric Co., Fairfield, CT, USA) using the same settings (70 kV, 15 mA). The size-1 radiographic films were used for the anterior regions, where size-2 radiographic films were used for the posterior regions to minimize the distortion of the films. All of the films were developed in a batch with an automatic developing machine (Periomat, Dürr Dental, Bietigheim-Bissingen, Germany) to reduce variability during the developing process.

All of the radiographs were subsequently scanned into a computer at 600 dpi with a flatbed scanner equipped with a transparency module (Agfa Studiostar, Agfa Gaevert, Mortsel, Belgium). The images were imported into software based on the Linux system. Selected sites were defined as regions of interest (ROI) on the radiographs, and the CADIA values were calculated for each ROI according to a previously reported formula [27]. The CADIA values were used to quantify changes in the alveolar bone and were presented as the net values of two standardized radiographic images, which were recorded at different time points [26].

GCF sampling was conducted according to the procedure reported by Jin et al. [33]. Briefly, for a subject tooth (same sites as the DSR study), after isolation with a cotton roll and the removal of the supragingival plaque, a standard filter GCF strip (Periopaper^®^ GCF strips, IDE Interstate, Amityville, NY, USA) was inserted into the gingival crevice or pockets until mild resistance was felt. The GCF strip was then left in place for 30 s. The amount of GCF collected was represented by the electric conductance, which was measured immediately using a GCF meter (Periotron 8000, IDE Interstate). The electrode jaws of the GCF meter were cleaned and dried after each measurement with the machine reading set to zero. The GCF volumes of two periodontitis-free sites were also followed in each participant, preferably from the same tooth type as the diseased tooth of interest. The GCF volumes from each sample site were calculated based on their electric conductance readings, which were measured using a Periotron 8000 GFC meter, as described previously [33]. Clinical data collection for the last participant was completed in December 2006.

### Data analysis

Data analysis was carried out using the statistics software: SPSS (SPSS 16.0, Chicago, IL, USA). Simple descriptive statistics were used to summarize the variables studied. Variations in demographic data and smoking habits between control and test groups were assessed by unpaired t tests. Variations in CM signs between control and test groups were assessed by Fisher’s exact test. Change of participants’ CM syndrome over time was assessed by Chi square test for trend.

As the normality (Kolmogorow-Smirnoff test) and homoscedasticity assumptions (Levene test) of the following data appeared to be valid, clinical parameters, including changes in CADIA, PAL, PPD, Pl % and BOP % were analyzed by repeated measures analysis of variance (ANOVA) with Dunnett’s post-test. Significance level was set as α < 0.05.

## Results

Eight of the 40 participants were considered ineligible by the CM practitioner because their CM signs and symptoms did not fit with the inclusion criteria. A total of 40 male smokers with chronic periodontitis were screened, and 32 met the inclusion criteria and were arbitrarily allocated (Fig. 2). Seventeen participants were assigned to receive PH001 (placebo), with the remaining 15 being assigned to receive PH002 (mYJ). A total of 7 (21.9 %) participants dropped out from the trial during the study period: 2 (11.8 %) and 3 (20.0 %) participants withdrew from the placebo and CM groups, respectively, because they believed that they had recovered already, while 1 participant from each group did not complete the trial because they left Hong Kong (Fig. 2).

**Fig. 3:**
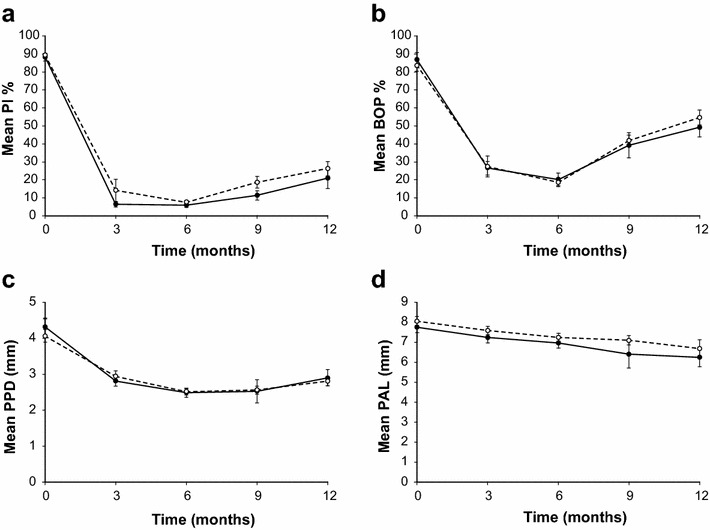
Mean (±SD) full-mouth periodontal parameters of participants from baseline to 12 months following periodontal treatment commencement. **a** Pl %, **b** BOP %, **c** PPD, **d** PAL, *Dashed line* placebo group; *solid line* mYJ group. No difference observable regarding mean Pl %, BOP % and PPD between test and control groups at any follow up time point while significant reduction was discernible regarding these three clinical parameters from base-line to all follow ups (**a**–**c**, *P* < 0.01, repeated measures ANOVA with Dunnett’s post-test). No difference observable in mean PAL between test and control groups at any time point and between baseline and all four time points for the test group, or up to 9 months for control group, while significant reduction of mean PAL from baseline was observable at month-12 in control group (*P* < 0.01, repeated measures ANOVA with Dunnett’s post-test)

The baseline characteristics of the participants are summarized in Table 2. No significant differences were observed between the two groups. The full-mouth periodontal parameters collected for all of the participants over the entire study period are shown in Fig. 3a–d. The results revealed that both groups showed improved plaque control and gingival inflammation (reflected by BOP % reduction) at 3 and 6 months post treatment commencement. However, the levels of plaque and the clinical signs of periodontal tissue inflammation appeared to increase beyond this point in both groups. The mean PPD and PAL values improved and became stable at 6–9 months, although the mean PPD value showed a tendency towards the baseline level at 12 months. No differences were observed in the mean Pl %, BOP % and PPD values between the test and control groups at any of the follow-up time points. Furthermore, the changes observed in the mean PAL over time were not significant for either of the groups compared with the baseline, except at 12 months, where we observed a significant improvement in PAL compared with the baseline in the control participants (Fig. 3d).

**Table 2:** Demography, pre-treatment clinical and CADIA sites profiles of participants

Characteristic	Categories	Placebo (n = 14)	mYJ (n = 11)	Test	Statistics	*P* value
Age	mean ± SD	43.6 ± 7.6	49.0 ± 5.0	*t*	2.0317	0.0539
*Smoking habit (self-reported)*						
Baseline	Smoked years	22.4 ± 7.7	25.0 ± 8.1	*t*	0.8193	0.4210
	Cigarettes/day	20.0 ± 9.2	22.3 ± 8.2	*t*	0.6502	0.5220
	Pack-years	22.2 ± 11.0	30.2 ± 21.4	*t*	1.2140	0.2371
End of study	Cigarettes/day	18.6 ± 7.6	21.4 ± 7.8	*t*	0.9040	0.3754
*Full*-*mouth parameter*						
Standing teeth	mean ± SD	23.1 ± 3.5	21.4 ± 3.2	*t*	1.272	0.2166
Plaque %	mean ± SD	89.4 ± 4.4	88.3 ± 7.1	*t*	0.3991	0.6954
BOP %	mean ± SD	83.7 ± 11.9	86.8 ± 13.2	*t*	0.6085	0.5497
% PPD ≥ 5 mm	mean ± SD	42.7 ± 19.4	44.6 ± 20.1	*t*	0.2382	0.8140
% PAL ≥ 7 mm	mean ± SD	80.6 ± 13.1	73.3 ± 21.0	*t*	1.009	0.3290
*CADIA sites (2/participant)*						
Tooth type	Incisor	8/0 (8)^a^	6/1 (7)	χ^2^	2.487	0.2884
	Canine	7/5 (12)	3/2 (5)			
	Premolar	4/4 (8)	5/5 (10)			
Plaque %		100	100			
BOP %		90.8	96.1	χ^2^	1.255	0.2626
PPD	mean ± SD	6.17 ± 1.68	6.33 ± 1.23	*t*	0.592	0.5598
PAL	mean ± SD	9.86 ± 2.23	9.02 ± 1.53	*t*	1.066	0.2977

*BOP* bleeding on probing, *PAL* probing attachment level, *PPD* probing pocket depth

^a^Upper/lower teeth (total)

All of the participants exhibited good tolerance to the study medicines, with none of the participants reporting any adverse effects. The overall compliance with the dose regimen was also good. According to the compliance sheets, all of the participants completed their course of herbal medicine, except for one participant who failed to attend the scheduled appointment and was therefore unable to collect the prepared medicine on time. In this particular case, the participant missed 2 weeks of CM medication during the third month. This participant was subsequently determined to be a control group participant, and was allowed to remain in the study.

**Fig. 4:**
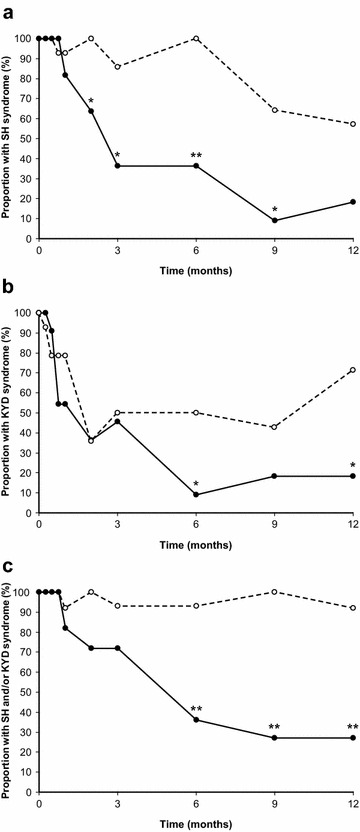
Proportion of participants with CM *stomach*-*heat* syndrome and/or *kidney*-*yin deficiency* over the study period. **a** *Stomach*-*heat* (SH) syndrome, **b** *Kidney*-*yin deficiency* (KYD), **c** combined *stomach*-*heat* and *kidney*-*yin deficiency*, *Dashed line* placebo group; *solid line* mYJ group. *P < 0.05, **P < 0.001, control vs. test group at corresponding time point, Fisher exact test. Significant improvement of *stomach*-*heat*, *kidney*-*yin deficiency* alone in test or placebo group or *stomach*-*heat* and/or *kidney*-*yin deficiency* in test group over time was observed (Chi square test for trend, *P* < 0.001); no such improvement could be observed in control group when combined *stomach*-*heat* and/or *kidney*-*yin deficiency* was followed over time (**c**)

The efficacy of the CM was noted (Fig. 4). The determination of SH syndrome can be subjective because of variations in the severity of the swelling and gum pain, thirst, halitosis, apatite, constipation and *long bi* (*short urine*) or dribbling urination [34]. The determination of KYD syndrome can also be subjective because of variations in the severity of the soreness of waist, backache, hot flushes and night sweating (*gu zheng* or *steaming bone)*, mobile teeth, hair loss, dryness of the mouth and throat, dizziness, tinnitus and deafness, wasting, insomnia, forgetfulness, while CM tongue and pulse signs are objectively accessed (Additional file 2: Table S1). The administration of a placebo during the non-surgical periodontal therapy led to the remission of KYD or SH syndrome in no more than half of the control group participants as early as 2 or 9 months after the commencement of the therapy, respectively. However, the use of mYJ as an adjunctive therapy for the treatment of non-surgical periodontal led to the remission of KYD and SH syndrome in approximately 80 % of the participants evaluated in this trial (Fig. 4). These effects were particularly prominent at 6 and 9 months post-treatment commencement for KYD and SH syndrome, respectively, and remained low at 12 months. Notably, only 1 out of the 14 participants in the control group showed total remission for the CM syndrome of SH/KYD at the end of the study, whereas complete CM syndrome resolutions were observed in 8 out of the 11 participant in the mYJ treatment group (Fig. 4c).

**Fig. 5:**
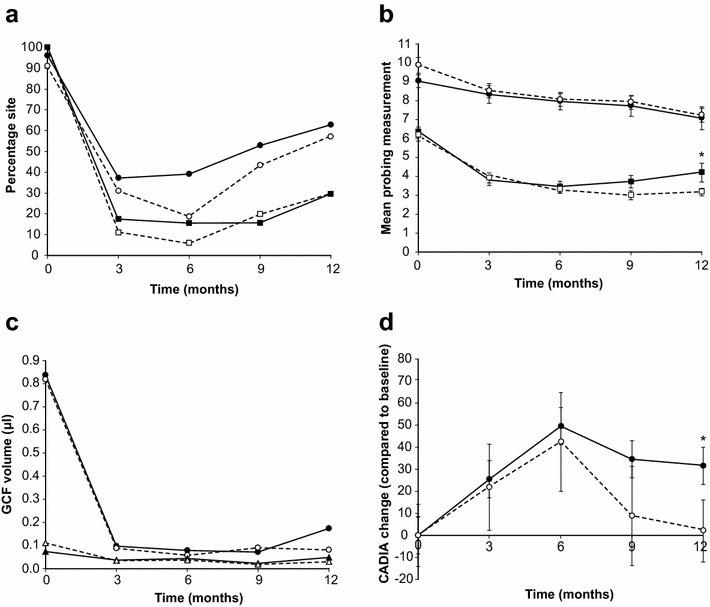
Mean (±SD) periodontal profiles of periodontitis sites of interest. **a** Pl % (*squares*) and BOP % (*circles*), **b** PPD (*squares*) and PAL (*circles*), **c** Gingival crevicular fluid (GCF) volume (µL/30 s) of periodontitis-free (*triangles*) or periodontits (*circles*) sites, and **d** change in CADIA values over the study period. *Dashed line* placebo group; *solid line* mYJ group. Significant difference in CADIA value changes between periodontitis sites in placebo group compared with mYJ group at 12 months (*P = 0.025, repeated measures ANOVA with Dunnett’s post-test)

Data pertaining to the selected periodontitis sites evaluated throughout this study are summarized in Fig. 5. Plaque control improved considerably at these periodontitis sites after the therapy, although the reoccurrence of some plaque was observed at the end of the study (i.e., at 12 months). A similar outcome was also observed for the percentage of sites with BOP and PAL. It is noteworthy that the mean depth of selected pockets sites in the mYJ group appeared significantly greater than that of the placebo group (*P* = 0.02, unpaired *t* test, Fig. 5a, b). The GCF volumes (µL/30 s paper strip sampling) of the selected sites remained low after the treatment process (Fig. 5c), with the corresponding GCF volumes of the periodontitis-free sites from the reference teeth on the same participant remained at a low level of 0.018–0.074 µL at every time point. Significant improvements were observed in the changes in the mean CADIA values at selected sites in the mYJ group participants at 12 months (Fig. 5d). Similar improvements were not observed in the control group.

## Discussion

Research towards the use of CM as adjunctive therapies in contemporary periodontal treatment appears to progressing slowly [17]. The symptoms of CM syndrome in patients suffering with periodontitis include *stomach*-*heat*, *kidney*-*yin* deficiency and weakness in the *qi* and *blood* [15, 35, 36]. Of the male smokers diagnosed with chronic periodontitis prior to this study, 80 % were also diagnosed with SH and KYD syndrome and were consequently invited to participate in our clinical trial (Fig. 2). Zhang et al. [37] reported that 27.8 % of the participants in their cohort study of 26 mild, 41 moderate and 23 severe conveniently recruited periodontitis participants (50 % female, unknown smoking habit) were diagnosed to have SH and KYD. The results of a subsequent study by the same group based on a similar group of conveniently recruited periodontitis patients (52.5 % female, unknown smoking habit) revealed that half of the participants experienced *kidney deficiency* syndrome [35]. Both of these studies showed a lower prevalence of SH and KYD than our current cohort. This difference was attributed to the fact that it was relatively easy to recruit and secure our trial sample size, especially male smokers with severe chronic periodontitis. However, a more in depth population study would be required to fully establish the relationship between severe chronic periodontitis and SH and/or KYD.

This study was therefore deigned to evaluate the effects of mYJ on the proximal periodontal radiographic alveolar bone density using the CADIA values as the primary dependent variable following therapy. In line with results from previous trials involving smokers treated periodontally according to a variety of different approaches [9, 23, 25], we did not expect to see significant differences in the non-radiographic periodontal clinical parameters like PPD reduction or PAL gain in the current small sample of smokers followed, instead CADIA was chosen as the dependent variable.

In general, despite having severe periodontitis, the smokers in the current study exhibited favorable initial periodontal healing in response to non-surgical periodontal therapy, as exemplified by the reduction in their PPD and PAL, which were similar to those reported previously [9]. The favorable clinical responses observed in this case were comparable with those reported in previous studies regarding non-surgical therapy in smokers [9, 38, 39].

Similar to the results of an earlier report [40], our control group of smokers exhibited only minor changes in their amount of radiographic bone density at 12 months after the commencement of non-surgical periodontal therapy compared with the baseline. In contrast, the test participants appeared to maintain the initial bone density gained after the non-surgical periodontal therapy (Fig. 5d). mYJ could therefore maintain the radiographic bone density gained after the non-surgical periodontal therapy for up to 12 month after the commencement of treatment. The control medication, however, did not maintain the radiographic bone density of the smokers 11 months after the periodontal therapy.

An un-blinded study by Luo et al. [41] used a 1 month, twice-daily CM recipe containing 12 different ingredients, including *gypsum fibrosum*, *Radix et Rhizoma Rehmanniae*, *Rhizoma Anemarrhenae* and *Cortex Phellodendri* as an adjunctive therapy in a 3-month clinical trial involving non-surgical periodontal therapy on twenty 40–50-year-old females (test group). The control group received non-surgical periodontal therapy without any placebo agent. The authors did not profile the CM status of their participants before and after the study period. Except for a lower BOP % value in the test group at 3 months, the changes in the PAL and PPD values between the two groups were not significant over the study period. One earlier study compared the twice-daily oral administration of a 4 mg dose of *Guchi* pills over 3 months (n = 50) with the four-times daily administration of an oral 200 mg dose of spiramycin over 5 days (n = 40) as adjunctive agents to non-surgical periodontal therapy [37]. *Guchi* pill contains 18 different ingredients, including *Radix et Rhizoma Rehmanniae, Radix Achyranthis Bidentatae* and *Rhizoma Drynariae*, which are three of the eight active ingredients of mYJ. The study monitored the participants for 24 months after the initiation of the study (i.e., the baseline). The authors reported that participants in the *Guchi* pills and spiramycin groups improved considerably. The 90 participants in this study consisted of a mixture of aggressive and chronic periodontitis patients with mild-to-severe disease at presentation and varied CM syndromes at baseline, including 28 % with SH and KYD. Unfortunately the authors presented limited clinical information, making it difficult to establish the true efficacy of the CM preparation or the spiramycin as adjunctive agents in non-surgical periodontal treatment.

With reference to the literature, purified or enriched extracts from the ingredients of the mYJ preparation used in this study have been independently reported to positively affect bone density in laboratory animals and cell cultures. For example, *Rehmanniae* has been reported to stimulate the proliferation of osteoblasts [42]; *Rhizoma Anemarhenae* [43] and *Drynariae Rhizoma* [44] have been reported to promote bone formation; and *Achyranthis bidentatae* [45] and *Puerariae radix* [46] have been reported inhibit bone loss. The remaining ingredients of mYJ including *Phellodendri cortex* and *Ophiopogonis* root have been reported to attenuate bacterial lipopolysaccharide-induced inflammation [47] and protect frog palatal epithelial cells against sodium metabisulphite induced damage [48], respectively. The major alkaloid component of *Phellodendri cortex*, berberine, has been reported to modulate the post-transcriptional regulatory mechanism of the iNOS gene, inhibit the activity of COX-2 and reduce the production of PGE_2_ in LPS-stimulated mouse macrophages [49]. *Cortex Phellodendri* and rhizome *Rehmanniae* have been shown to inhibit *Porphyromonas gingivalis* in vitro [50]. The major ingredients in mYJ could potentially attenuate periodontal inflammation and promote bone healing.

The control flavoring agent: *Momordica Charantia* despite having CM *heat* clearing alleviation of dental pain plus hypoglycemic effects [51], was not reported to possess bone cell stimulation functions. The dose and duration use of *Momordica Charantia* administered could not clear the SH of all the periodontitis-affected male smokers.

The fact that 64–73 % of the mYJ group participants remained free from SH and/or KYD syndromes after the 6-month recall in the study indicated that mYJ was effective in alleviating the corresponding syndrome in these smoking periodontitis individuals. The reason why 3-to-4 mYJ group participants continued exhibiting SH and/or KYD syndrome, remains to be elucidated.

The mYJ formula might maintain the improved alveolar bone density from the immediate post-treatment stage up to 12 months, while the positive hard tissue effects detectable on radiographs at selected sites with placebo adjunctive to non-surgical periodontal therapy could only be maintained over the first 6 months. By 9 months, radiographic bone densities of control sites had reverted to the pre-treatment levels.

## Conclusions

The adjunctive use of mYJ preserved the post-treatment increases in the radiographic alveolar bone density at the study sites and led to an overall improvement in SH and KYD compared with the controls.

## Additional files

10.1186/s13020-016-0111-z Ethical approval, explanatory note and consent form.
10.1186/s13020-016-0111-z CM differential diagnosis regarding: syndrome of deficient of *kidney-yin* or *stomach-heat.*

## Abbreviations

BOP: bleeding on probing
CADIA: computer-assisted densitometric image analysis
CM: Chinese medicine
DSR: digital subtraction radiography
GCF: gingival crevicular fluid
KYD: *kidney*-*yin deficiency*
mYJ: modified *Yunu*-*Jian*
Pl %: plaque percentage
PPD: probing pocket depth
PAL: probing attachment level
ROI: regions of interest
SH: *stomach*-*heat*

## References

[1] Williams RC (2008). Understanding and managing periodontal diseases: a notable past, a promising future. J Periodontol.

[2] Ng SKS, Leung WK (2006). A community study on the relationship between stress, coping, affective dispositions and periodontal attachment loss. Community Dent Oral Epidemiol.

[3] Page RC, Kornman KS (2000). The pathogenesis of human periodontitis: an introduction. Periodontol.

[4] Johnson GK, Hill M (2004). Cigarette smoking and the periodontal patient. J Periodontol.

[5] Zee KY (2009). Smoking and periodontal disease. Aust Dent J.

[6] Grossi SG, Zambon JJ, Ho AW, Koch G, Dunfort RG, Machtei EE, Norderyd OM, Genco R (1994). Assessment of risk for periodontal disease. I. Risk indicators for attachment loss. J Periodontol.

[7] Jin L, Wong KY, Leung WK, Corbet EF (2000). Comparison of treatment response patterns following scaling and root planing in smokers and non-smokers with untreated adult periodontitis. J Clin Dent.

[8] Darby I, Hodge PJ, Riggio MP, Kinane DF (2005). Clinical and microbiological effect of scaling and root planing in smoker and non-smoker chronic and aggressive periodontitis patients. J Clin Periodontol.

[9] Wan CP, Leung WK, Wong MC, Wong RM, Wan P, Lo EC, Corbet EF (2009). Effects of smoking on healing response to non-surgical periodontal therapy: a multilevel modelling analysis. J Clin Periodontol.

[10] Machtei EE, Hausmann E, Schmidt M, Grossi SG, Dunford R, Schifferle R, Munoz K, Davies G, Chandler J, Genco RJ (1998). Radiographic and clinical responses to periodontal therapy. J Periodontol.

[11] Flaws B, Sionneau P (2005). The treatment of modern western diseases with Chinese Medicine: a textbook and clinical manual.

[12] Gold SI (1985). Periodontics. The past. Part (I). Early sources. J Clin Periodontol.

[13] Wang T. Waitai Miyao. Beijing: People’s Medical Publishing House; 752. (Reprinted 1955) (**In Chinese**).

[14] Zhao W, Zhao Q (2009). The overview of the prevention and treatment of dental disease in ancient China. Zhonghua Yi Shi Za Zhi.

[15] Zhang J: Jingyue Quan Shu. Beijing: People’s Medical Publishing House; 1624. (Reprinted 1991) (**In Chinese**).

[16] Wu Y: Bencao Congxin. Shanghai: Shanghai scientific and Technical Publishers; 1886. (Reprinted 2002) (**In Chinese**).

[17] Cao CF, Sun XP (1998). Herbal medicine for periodontal diseases. Int Dent J.

[18] Xu ZH (2006). Current status and prospect of prevention and treatment of oral diseases by integrative medicine. Chin J Integr Med.

[19] Wong RWK, Rabie ABM (2006). Traditional Chinese medicines and bone formation—a review. J Oral Maxillofac Surg.

[20] Zhang P, Dai KR, Yan SG, Yan WQ, Zhang C, Chen DQ, Xu B, Xu ZW (2009). Effects of naringin on the proliferation and osteogenic differentiation of human bone mesenchymal stem cell. Eur J Pharmacol.

[21] Liu ZW (2007). Essentials of Chinese Medicine.

[22] Badersten A, Nilveus R, Egelberg J (1984). Effect of nonsurgical periodontal therapy. II. Severely advanced periodontitis. J Clin Periodontol.

[23] Angaji M, Gelskey S, Nogueira-Filho G, Brothwell D (2010). A systematic review of clinical efficacy of adjunctive antibiotics in the treatment of smokers with periodontitis. J Periodontol.

[24] Kerdvongbundit V, Wikesjö UME (2003). Effect of triclosan on healing following non-surgical periodontal therapy in smokers. J Clin Periodontol.

[25] Krohn-Dale I, Bøe OE, Enersen M, Leknes KN (2012). Er:YAG laser in the treatment of periodontal sites with recurring chronic inflammation: a 12-month randomized, controlled clinical trial. J Clin Periodontol.

[26] Lai SML, Zee K-Y, Lai MK, Corbet EF (2009). Clinical and radiographic investigation of the adjunctive effects of a low-power He-Ne laser in the treatment of moderate to advanced periodontal disease: a pilot study. Photomed Laser Surg.

[27] Brägger U, Pasquali L, Rylander H, Carnes D, Kornman KS (1988). Computer-assisted densitometric image analysis in periodontal radiography. A methodological study. J Clin Periodontol.

[28] Hausmann E (2000). Radiographic and digital imaging in periodontal practice. J Periodontol.

[29] Woo BM, Zee KY, Chan FH, Corbet EF (2003). In vitro calibration and validation of a digital subtraction radiography system using scanned images. J Clin Periodontol.

[30] Jiang HL, She B, Liu W, Mao B, Zhang JY (2016). Efficacy and safety of Qi-Wei-Qing-Yan aerosol in treatment of acute pharyngitis (*lung*-*stomach* excess-heat syndrome): study protocol for a randomized controlled trial. Trials.

[31] Kadam P, Bhalerao S (2010). Sample size calculation. Int J Ayurveda Res.

[32] Janssen PTM, van Palenstein Helderman WH, van Aken J (1989). The effect of in vivo-occurring errors in the reproducibility of radiographs on the use of the subtraction technique. J Clin Periodontol.

[33] Jin L, Yu C, Corbet EF (2003). Granulocyte elastase activity in static and flow gingival crevicular fluid. J Periodontal Res.

[34] Zhu W, Fan Z, Liu G, Yan J, Zhong T, Zheng W, Wang R, Wang C (2014). Symptom clustering in chronic gastritis based on spectral clustering. J Tradit Chin Med.

[35] Ma Z-J, Zhang J-Z (1993). Changes of serum zinc level of periodontitis patients with kidney deficiency. Chin J Integr Med.

[36] Xu ZX, Peng XZ, Ma HM, Wang X, Liu GX (1995). Relationship between kidney insufficiency and some endocrine hormones in periodontitis patients. Zhonghua Kou Qiang Yi Xue Za Zhi.

[37] Zhang J-Z, Yang X-X, Tong Y-H (1992). Clinical study of combined Gu Chi Wan and spiramycin in the treatment of periodontal disease. Zhongguo Zhong Xi Yi Jie He Za Zhi.

[38] Renvert S, Dahlén G, Wikström M (1998). The clinical and microbiological effects of non-surgical periodontal therapy in smokers and non-smokers. J Clin Periodontol.

[39] Palmer RM, Matthews JP, Wilson RF (1999). Non-surgical periodontal treatment with and without adjunctive metronidazole in smokers and non-smokers. J Clin Periodontol.

[40] Preshaw PM, Heasman PA (2005). Periodontal maintenance in a specialist periodontal clinic and in general dental practice. J Clin Periodontol.

[41] Luo LJ, Gu JJ, Liu XF, Yu J, Shu R (2004). Treatment of chronic periodontitis with traditional Chinese medicine. J Pract Stomatol..

[42] Oh KO, Kim SW, Kim JY, Ko SY, Kim HM, Baek JH, Ryoo HM, Kim JK (2003). Effect of *Rehmannia glutinosa Libosch* extracts on bone metabolism. Clin Chim Acta.

[43] Nian H, Qin LP, Chen WS, Zhang QY, Zheng HC, Wang Y (2006). Protective effect of steroidal saponins from rhizome of *Anemarrhena asphodeloides* on ovariectomy-induced bone loss in rats. Acta Pharmacol Sin.

[44] Wong RW, Rabie B, Bendeus M, Hägg U (2007). The effects of *Rhizoma Curculiginis* and *Rhizoma Drynariae* extracts on bones. Chin Med.

[45] He CC, Hui RR, Tezuka Y, Kadota S, Li JX (2010). Osteoprotective effect of extract from *Achyranthes bidentata* in ovariectomized rats. J Ethnopharmacol.

[46] Wang X, Wu J, Chiba H, Yamada K, Ishimi Y (2005). *Puerariae radix* prevents bone loss in castrated male mice. Metabolism.

[47] Mao YF, Li YQ, Zong L, You XM, Lin FQ, Jiang L (2010). Methanol extract of *Phellodendri cortex* alleviates lipopolysaccharide-induced acute airway inflammation in mice. Immunopharmacol Immunotoxicol.

[48] O’Brien DW, Morris MI, Lee MS, Tai S, King M (2004). *Ophiopogon* root (*Radix Ophiopogonis*) prevents ultra-structural damage by SO_2_ in an epithelial injury model for studies of mucociliary transport. Life Sci.

[49] Lee DU, Kang YJ, Park MK, Lee YS, Seo HG, Kim TS, Kim CH, Chang KC (2003). Effects of 13-alkyl-substituted berberine alkaloids on the expression of COX-II, TNF-α, iNOS, and IL-12 production in LPS-stimulated macrophages. Life Sci.

[50] Wong RW, Hägg U, Samaranayake LP, Yuen MKZ, Seneviratne CJ, Kao R (2010). Antimicrobial activity of Chinese medicine herbs against common bacteria in oral biofilm. A pilot study. Int J Oral Maxillofac Surg.

[51] Basch E, Gabardi S, Ulbricht C (2003). Bitter melon (*Momordica charantia*): a review of efficacy and safety. Am J Health Syst Pharm.

