# Supplementary material for: Adjunctive use of modified Yunu-Jian in the non-surgical treatment of male smokers with chronic periodontitis: a randomized double-blind, placebo-controlled clinical trial
Source: Chin Med. 2016 Sep 20;11:40. doi: 10.1186/s13020-016-0111-z (PMC5028984; doi:10.1186/s13020-016-0111-z)
Supplement: Supplementary file 2 — 10.1186/s13020-016-0111-z CM differential diagnosis regarding: syndrome of deficient of kidney-yin or stomach-heat. [file 13020_2016_111_MOESM2_ESM.docx]

**Supplementary Table 1 CM differential diagnosis regarding: syndrome of deficient of *kidney-yin* or *stomach-heat***

|  | **CM differential diagnosis** [S1, S2] | |
| --- | --- | --- |
|  | ***Kidney-yin* deficiency** | ***Stomach-heat*** |
| Oral symptoms |  |  |
| Teeth mobility | Yes | No |
| Gum colour | Ruddy (reddish) | Red |
| Gum condition | Ulcer with ruddy swelling periphery | Swelling and painful |
| Tooth root exposure | Yes | No |
| Gum bleeding | Yes | Yes |
| Pus | No | Yes |
|  |  |  |
| Systemic General symptoms | | |
| Dizziness and tinnitus | Yes | No |
| Soreness of waist, backache | Yes | No |
| Hectic fever and night sweat | Yes | No |
| Palm and foot hot | Yes | No |
| Halitosis | No | Yes |
| Thirst | No | Yes |
| Drinks preference | No | Prefer cold drinks |
| Forgetfulness | Yes | No |
| Appetite | Normal | Polyphagia and bulimic |
| Urination and Defecation | Normal | Constipation |
|  |  |  |
| Tongue and Pulse sign | | |
| Tongue sign | Reddened tongue with thin coating | Reddened tongue with thick yellow coating |
| Pulse sign | Small and rapid | Large or slippery rapid |

**References**

S1. Wang D: Kouqiang ke gaishu. In: Wang D, Wang S [Editors]: Zhongyi Erbiyanhou Kouqiang Kexue. People’s Medical Publishing House, Beijing: PR China. 1994, Chapter 4, pp. 125-58. [In Chinese]

S2. Wang Y [Chief Editor]: Zhongyi Erbiyanhou Kouqiang Kexue. People’s Medical Publishing House, Beijing: PR China. 2001. [In Chinese]
