# Supplementary material for: Adjunctive use of modified Yunu-Jian in the non-surgical treatment of male smokers with chronic periodontitis: a randomized double-blind, placebo-controlled clinical trial
Source: Chin Med. 2016 Sep 20;11:40. doi: 10.1186/s13020-016-0111-z (PMC5028984; doi:10.1186/s13020-016-0111-z)
Supplement: Supplementary file 1 — 10.1186/s13020-016-0111-z Ethical approval, explanatory note and consent form. [file 13020_2016_111_MOESM1_ESM.pdf]

THE UNIVERSITY OF HONG KONG

Faculty of Dentistry

The Prince Philip Dental Hospital,
34 Hospital Road, Hong Kong.
Telephone: 2859 0341
Fax: (852) 2517 0544

Ref.1/8/12d

January 8, 2004

Dr. K.Y. Zee
Faculty of Dentistry

Dear Dr. Zee,

Research protocol entitled: "Adjunctive effect of a Chinese herbal medicine in the treatment of advanced periodontal disease: A randomized controlled clinical trial"

I am pleased to confirm that approval has been granted for the above-named research protocol.

Yours sincerely,

Rupert Chan
Secretary
Faculty of Dentistry

cc: Chairman

THE UNIVERSITY OF HONG KONG

Faculty of Dentistry

The Prince Philip Dental Hospital,
34 Hospital Road, Hong Kong.
Telephone: (852) 2859 0341
Fax: (852) 2517 0544

August 21, 2014

Editor
The Chinese Medicine Editorial Team

Dear Editor,

Our Faculty Ethics Committee considered Dr. K.Y. Zee's research protocol entitled "Adjunctive effect of a Chinese herbal medicine in the treatment of advanced periodontal disease: A randomized controlled clinical trial" and granted ethical approval to this research protocol. The approval letter was issued by our former Faculty Secretary Mr. Rupert Chan on January 8, 2004.

2. For your information, our Faculty Ethics Committee was a committee formally established by the Board of the Faculty of Dentistry to, *inter alia*, consider individual research protocols submitted to the Committee. It was the sole approving authority for ethical approval of research protocols in the Faculty of Dentistry prior to the disestablishment of this Committee in June 2006.

Yours sincerely,

Vivien Chan

(Ms) Vivien Chan
Acting Secretary
Faculty of Dentistry

香　港　大　學
THE UNIVERSITY OF HONG KONG

Effects of modified Yunu Jian, a Traditional Chinese Medicine formula, in non-surgical periodontal treatment of smokers with periodontitis

Explanatory notes for subjects (English)

1. Periodontal disease is caused by germs (dental plaque) inside your mouth and the reaction of the germs to your own body defense system.
2. The approach of Tradition Chinese Medicine (TCM) in treating teeth and gum problems emphases the balance of different systems and organs inside the body, such as Spleen and Kidney which are usually related to the periodontal tissues. In modern dentistry, conventional treatment is based mainly on the removal of the causative agent above and below the gum margin and the prevention of its deposition. There are limited data on the effect of combining TCM and conventional therapy in treating gum diseases. Hence, you are invited to participate in this study where we plan to investigate whether there is any additional effect of TCM in the treatment of periodontal disease.
3. The treatment will involve:
   - Comprehensive examination of your periodontal diseases
   - All of you will be examination by an experienced Chinese Medicine Practitioner and some of you will be selected to be the test group in which a Chinese Herbal Medicine will be provided for your gum disease and the rest will receive a placebo formula
   - Oral hygiene instruction
   - Removal of all deposits (located above and below your gum) on your teeth by conventional method
   - Periodic examination and maintenance
4. The Chinese herbal medicine preparation used in this study is modified from an ancient formula and is produced by Eu Yan Sang International Ltd using modern technology. This formula was developed according to TCM principles and has been recommended to those patients
5. Your gum will be examined before and after the periodontal treatment and during the maintenance period. All the examination procedures are not invasive and will not cause any discomfort or pain.
6. You may choose to leave the study without prejudice to the planned treatment.

Thank you for your co-operation.

Dr K.Y. Zee

Dr. J. Ho

Miss P.S. Chan (Registered Chinese Medicine Practitioner)

香港大學牙醫學院牙周病學及公共衞生學
Periodontology & Public Health, Faculty of Dentistry
3/F, Prince Philip Dental Hospital, 34 Hospital Road, Hong Kong.
TEL: (852) 2859 0301　　FAX: (852) 2858 7874

中藥及牙周病治療

給病人的研究細則說明

1. 牙周病是由口腔內的細菌（牙垢膜）及身體免疫系統對此等細菌的反應而形成。

2. 在中醫學治療牙齒和牙肉的問題上，通常是透過調理身體五臟六腑，例如腎和脾；而西方牙醫學上，治療牙周病的方法主要是清除牙齒上的細菌。到目前為止，在融合中藥及西方牙周病的治療方面還沒有太多的研究，故此我們現誠邀閣下參與此項研究，藉此對中草藥於牙周病的療效有更深的認識。

3. 所涉及的治療包括：
   - 對閣下的牙周病作出詳盡的檢查
   - 所有病人會接受中醫師的診斷而部份病人會被選入中草藥治療組
   - 口腔衛生指導
   - 清除所有在牙齒或牙腳上的牙石及牙垢膜
   - 定期檢查

4. 在此研究所用的中藥是根據古複方加減而成，由余仁生國際有限公司用現代化生產方法製成，用以協助治療牙周病。

5. 閣下的牙周將於治療前、後及定期檢查時作出詳盡的檢查。過程絕不會引致不適及痛楚。

6. 閣下有權隨時拒絕參與此研究而不影響閣下的治療。

多謝閣下參與。

徐君逸醫生
何晉陞醫生
陳沛思醫師謹啟

香港大學牙醫學院牙周病學及公共衛生學
Periodontology & Public Health, Faculty of Dentistry
3/F, Prince Philip Dental Hospital, 34 Hospital Road, Hong Kong.
TEL: (852) 2859 0301 FAX: (852) 2858 7874

香 港 大 學
THE UNIVERSITY OF HONG KONG

Effects of modified Yunu Jian, a Traditional Chinese Medicine formula, in non-surgical periodontal treatment of smokers with periodontitis

中藥及牙周病治療研究

Consent Form

同意書

I, __________________________ (I/D No.:__________ PPDH No.:__________), give consent to participate in this study. The experimental procedures have been explained to me and I understand what they entail. I further understand that I have the right to withdraw from the study without any questioning or prejudice to my future treatment.

本人已了解關於此項牙周病之研究細則，而同意參與此項研究及接受檢查和治療。同時本人明白有權隨時拒絕參與此項研究，而不影響本人應接受之治療。

Signed (簽署): ____________________________

Date (日期): _______________________

Witnessed (見證人): __________________________

香港大學牙醫學院牙周病學及公共衞生學
Periodontology & Public Health, Faculty of Dentistry
3/F, Prince Philip Dental Hospital, 34 Hospital Road, Hong Kong.
TEL: (852) 2859 0301 FAX: (852) 2858 7874
